# Uncloaking the black-box: the need for explainable artificial intelligence in clinical microbiology and infectious diseases applications

**DOI:** 10.3389/fpubh.2026.1776922

**Published:** 2026-04-02

**Authors:** Sreya Pulakkat Warrier, Venkatesh Narasimhan, Eline Meijer, Yukino Gütlin, Oliver Nolte, Balaji Veeraraghavan, Adrian Egli

**Affiliations:** 1 Department of Clinical Microbiology, Christian Medical College and Hospital, Vellore, India; 2 Institute of Medical Microbiology, University of Zurich, Zurich, Switzerland

**Keywords:** antimicrobial resistance, artificial intelligence, deep learning, explainable AI/XAI, genomics, infectious diseases, lime, machine learning

## Abstract

Antimicrobial resistance and emerging infectious diseases remain significant challenges for global health, driving a need for advanced technological solutions. Artificial Intelligence (AI) expanded opportunities in clinical microbiology, infectious diseases, and public health by harnessing vast, structured datasets. Despite impressive analytical capabilities, the clinical integration of AI-based applications is hindered by its opacity. The “black-box” aspect undermines adoption into healthcare workflows. Explainable AI (XAI) methods, including intrinsically interpretable models and post-hoc interpretability tools, such as SHAP, LIME, and Grad-CAM, can address these transparency challenges. This narrative review is intended to be a primer for the interested clinician. It systematically evaluates recent advancements in XAI in the context of clinical applications for clinical microbiology, infectious diseases, and public health. We further discuss the ethical and regulatory landscape shaping AI adoption, including the critical role of open, quality-controlled data, robust performance metrics, and clear interpretability to ensure safe and effective clinical implementation. Lastly, we propose future directions, emphasizing interdisciplinary collaboration, international data-sharing initiatives, and tailored AI literacy training to facilitate trustworthy, equitable, and impactful use of AI in clinical microbiology and infectious diseases.

## Introduction

**Figure 1:**
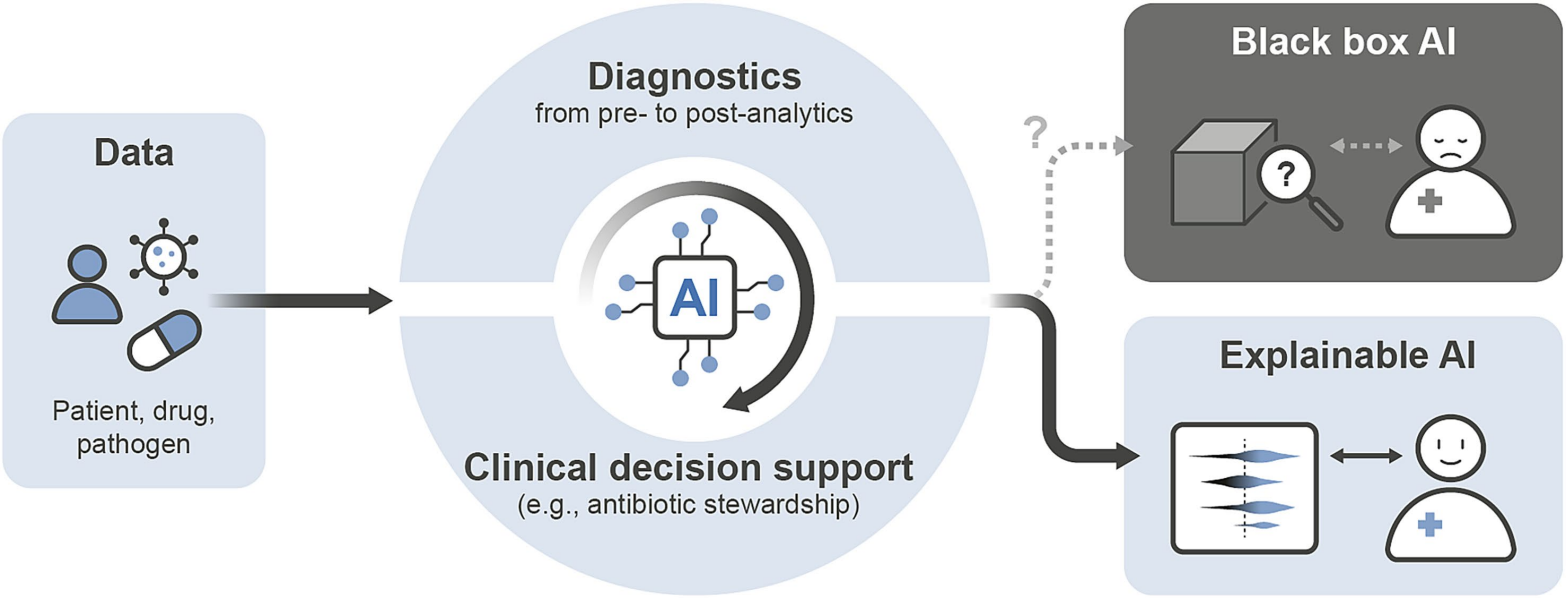
Integration of artificial intelligence (AI) into diagnostic and antimicrobial stewardship workflows. Clinical and laboratory data encompassing patient characteristics, drug information, and pathogen attributes serve as foundational input adhering to FAIR (Findable, Accessible, Interoperable, Reusable) data principles. The AI system operates across multiple stages of diagnostic decision-making, including pre-analytics (guiding appropriate diagnostic tests), analytics (identifying pathogens, performing antimicrobial susceptibility testing [AST], and outbreak detection), post-analytics (interpreting results and suggesting subsequent clinical actions), and decision support (enhancing antibiotic stewardship). The output of AI models can follow two distinct pathways: (i) “Black box” models, which lack transparency and may confuse clinicians (upper box), and (ii) explainable AI models, providing interpretable results that empower physicians, facilitating clear decision-making and confident clinical actions (lower box).

Around the globe, antimicrobial resistant and virulent pathogens continue to drive morbidity, mortality, and escalating healthcare costs, pose ongoing public health challenges, and impact modern medicine (1, 2). Despite transformative advances in hygiene, antibiotics, and vaccines, infectious diseases remain a major cause of childhood mortality worldwide, but affect all age groups, ranking alongside cardiovascular diseases and cancer (3). Recent surveillance efforts during the COVID19 pandemic (4), Mpox (5), and *Corynebacterium diphtheriae* (6) outbreaks highlight the crucial role of open data in diagnostics, infection control, and enhancing insights into pathogen biology (7, 8). The exponential growth of structured, open healthcare and laboratory datasets, and high-performance computing has expanded opportunities for artificial intelligence (AI)-founded applications in clinical microbiology, infectious diseases, and public health (9, 10). To effectively implement these technologies, a robust, infectious disease-focused data ecosystem is necessary. Adherence to high quality, curated, and FAIR [Findable, Accessible, Interoperable, and Reusable (7, 11)] data principles is essential to maximise their utility for domain-specific clinical and research purposes. In our case, AI models trained on large-scale microbiological diagnostic data can be adapted to a wide range of tasks, even though they were not explicitly trained for each specific application (12, 13). However, a major critical aspect of the ongoing technological revolution in medicine is the lack of explainability of highly complex AI applications and their underlying models. This results in resistance to implement such algorithms in diagnostic and therapeutic workflows, especially when linked to human healthcare outcomes (Figure 1).

For this narrative review, we define AI as the capability of computer systems to perceive, reason, learn, plan, and predict in ways analogous to those of medical specialists (14, 15). AI models that leverage historical data to identify patterns and predict, e.g., antimicrobial resistance directly from MALDI-TOF mass spectra (16), help to discover new antibiotics (17), support clinical decision making in, e.g., treatment of sepsis (18), or predict the outcome of sepsis (19, 20). Increasingly, AI not only expands medical knowledge but also significantly enhances our understanding of fundamental mechanisms in infection biology (21–23). In the following sections, we will summarize the status quo and discuss recent developments of explainable AI (XAI) with a focus on infectious diseases.

The studies were identified through a literature review covering January 2000 to July 2025 using combinations of keywords such as “microbiology,” “infectious diseases,” “pathogen,” “antimicrobial resistance,” “genomics,” “metagenomics,” “artificial intelligence,” “machine learning,” “deep learning,” “explainable AI/XAI,” “SHAP,” and “LIME” on PubMed/MEDLINE, Google Scholar and traditional Google searches, with a focus on papers published post-2021, particularly in 2024 and 2025.

## Fundamentals of artificial intelligence

AI encompasses a broad field dedicated to creating systems capable of emulating intelligent human behaviour without direct human intervention (15). AI’s origins trace back to the 1950s, with Alan Turing’s foundational work on machine intelligence and the 1956 Dartmouth Conference, which established AI as a scientific field aimed at replicating and surpassing human cognition. Recently, advanced large language models (LLMs) were brought into mainstream, highlighting their potential within diagnostic laboratories (24).

*Learning strategies*. AI models are highly effective in rapidly categorizing data. These models generate predictions by learning on prior experiences captured in data inputs and typically rely on supervised, unsupervised, or reinforcement learning strategies (14, 25). In *supervised learning*, the ground truth is known, and this is a typical teacher-student relationship. Supervised learning algorithms utilize labelled datasets to predict outcomes or classify inputs, e.g., Gram-staining categories and morphologies of bacteria in microscopy. In contrast, *unsupervised learnin*g operates without labelled data; methods such as K-means clustering and hierarchical clustering identify intrinsic data patterns without labelled inputs. These techniques, including anomaly detection methods and principal component analysis, are notably effective in biomarker discovery contexts, e.g., grouping of patients based on gene expression upon SARS-CoV-2 infection (26). Finally, *reinforcement learning* is a training strategy in which an agent learns an optimal sequence of actions by interacting with an environment and receiving scalar rewards that signals success or failure; the objective is to maximise the long-term discounted return, typically formalised as a Markov decision process (27). In the clinical domain, reinforcement learning is uniquely suited to sequential decision-making problems where actions taken now influence downstream patient outcomes, such as in sepsis management (18) or learning drug cycling policies to reduce evolution of antimicrobial resistance (28).

**Figure 2:**
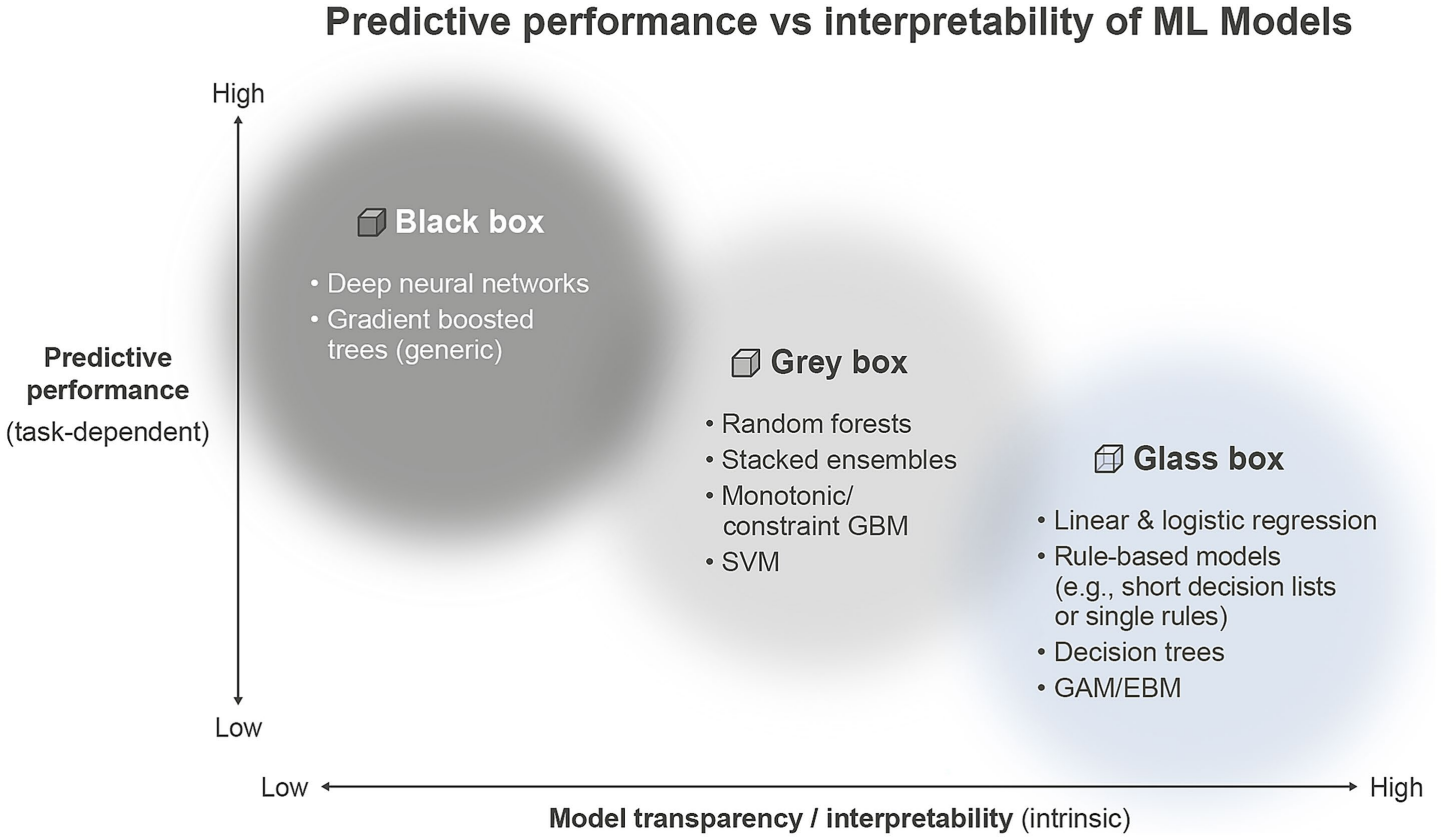
The accuracy and interpretability tradeoff in machine learning models. The figure illustrates the inverse relationship typically observed between model accuracy (*y*-axis) and interpretability (*x*-axis). Highly complex models such as deep neural networks (DNN), support vector machines with radial basis function kernels (SVM-RBF), and extreme gradient boosting (XGBoost) often achieve superior accuracy but at the expense of interpretability (“Black box” models, top-left). Intermediate models like random forests and fuzzy rule-based systems offer moderate interpretability and accuracy (“Grey box” models, middle). Simpler models such as decision trees, logistic regression, and linear regression provide high interpretability, making them preferable in contexts where transparent decision-making and explainability are critical, albeit often with lower accuracy (“Glass box” models, lower-right).

*Accuracy vs. interpretability*. Historically, symbolic AI programs from the 1950s–1970s inherently provided transparency through clear, expert-authored “if-then” rules. However, the shift towards statistical learning methods and neural networks in the 1990s improved predictive accuracy at the expense of interpretability, increasing complexity and obscuring decision-making logic (29). Often in AI models there is a trade-off between accuracy vs. interpretability (Figure 2).

*Traditional machine learning models*. Traditional machine learning (ML) methods refer to a set of well-established algorithms and models that are used to analyse and make predictions based on structured data. These methods typically rely on statistical techniques and mathematical models to learn patterns and relationships from data, often with a focus on interpretability and simplicity. Traditional ML methods involve more straightforward algorithms like linear regression, decision trees, support vector machines, k-nearest neighbours, random forests, and gradient boosting (30).

*Neural networks and deep learning.* The concept of neural networks has been around for decades but only gained substantial traction with the rise of increased computational power (31). Deep learning is a branch of AI in which data is processed through multiple layers of interconnected neurons. Each neuron is a simple computational unit that combines input values and passes the result forward. Together, these layers progressively extract more complex patterns from the data. For example, in image analysis, early layers may detect simple features such as edges or shapes, while later layers combine this information into more complex patterns, such as bacterial colony morphology. The complexity of these models is usually described by the number of layers and neurons (nodes), often summarized as the number of parameters. Different types of neural networks exist, referred to as different architectures, which differ in how their neurons are organized and how they process information. One type of such architecture is Recurrent Neural Network (RNN), specifically designed to handle sequential data. Unlike traditional neural networks that process data independently, RNNs have “memory” or hidden states that allow them to remember previous inputs, making them suitable for tasks like language translation, natural language processing, sentiment analysis, and time series forecasting. RNNs were used in the discovery of synthetic antimicrobial peptides (32). Another common architecture is Convolutional Neural Network (CNN), particularly well suited for image analysis. CNNs inherently integrate feature extraction processes, e.g., used for the identification of bacterial species through colony morphology on agar plates (33) and rapid Gram-stain characterization in positive blood cultures (34).

*Foundational models & multi-model models.* Foundation models are based on transformer architecture, which allow them to learn from vast amounts of data through a process called self-supervised learning. In the case of LLMs, the model predicts the next word in a sentence or sequence of data, enabling it to understand context and relationships without needing explicitly labelled data. LLMs have a broad spectrum of applications in clinical microbiology and infectious diseases (24), e.g., the interpretation of disk diffusion diameters to predict the underlying molecular antimicrobial resistance mechanisms (35). Additionally, multi-modal approaches that combine different types of inputs are increasingly being used to solve complex, multi-dimensional problems (12). These approaches are opening new avenues for research and clinical applications, especially in areas that require combining multiple data types, ultimately enabling AI systems to perform tasks that were previously considered too complex for conventional models.

*Accuracy vs. interpretability trade-off.* A critical ongoing debate involves balancing model interpretability with predictive accuracy [Figure 2, (36, 37)]. Simple, interpretable models like decision trees typically offer clear logic but are often surpassed in predictive performance by complex ensemble models. However, recent studies show interpretable models can achieve competitive performance even in critical clinical settings. For instance, a gradient-boosting model combined with SHapley Additive exPlanations (SHAP) values effectively predicted sepsis in emergency department triage, matching deeper neural networks in predictive performance, while simultaneously clarifying the influence of critical clinical factors such as heart rate increases and hypotension (38). The choice of model complexity must carefully weigh incremental accuracy gains against ethical and practical considerations, such as biases inherent in opaque models. For example, correcting predictive biases across socioeconomic groups may negate the slight accuracy advantage of complex models, making simpler, interpretable options safer, more transparent, and ethically preferable (39, 40). For these approaches, clear methods to assess their reliability and limits are essential. Despite their high predictive accuracy, the rapid evolution and complexity of AI models often result in opaque, “black-box” decision-making processes. This opacity fosters clinician skepticism and mistrust, limiting broader clinical adoption (41).

## Explainable AI

Explainability complements interpretability by clarifying reasons behind prediction. Using XAI bridges this transparency gap by offering understandable explanations that humans can interpret, trust, and troubleshoot. Enhanced explainability fosters clinician trust and facilitates safer incorporation into routine clinical practice (41).

*Strategies for enhanced interpretability.* Modern XAI methodologies enhance interpretability through three principal approaches: (i) Intrinsic interpretability with models, such as decision trees or rule-based classifiers, inherently limit complexity to maintain clear, transparent logic; (ii) post-hoc explanations; and (iii) hybrid architectures (29).

*Intrinsic interpretability*. Intrinsic interpretability refers to models that are inherently interpretable due to their design. These models, such as decision trees or rule-based classifiers, intentionally limit complexity to maintain clear and transparent decision-making logic. The principle of Occam’s Razor applies here, with simpler models that provide sufficient explanatory power often being preferable when their predictive performance is comparable to more complex alternatives (42). For example, sparse decision trees offer a level of clarity that can be especially beneficial in fields like malaria risk assessment, where the decision process needs to be actionable (43).

**Figure 3:**
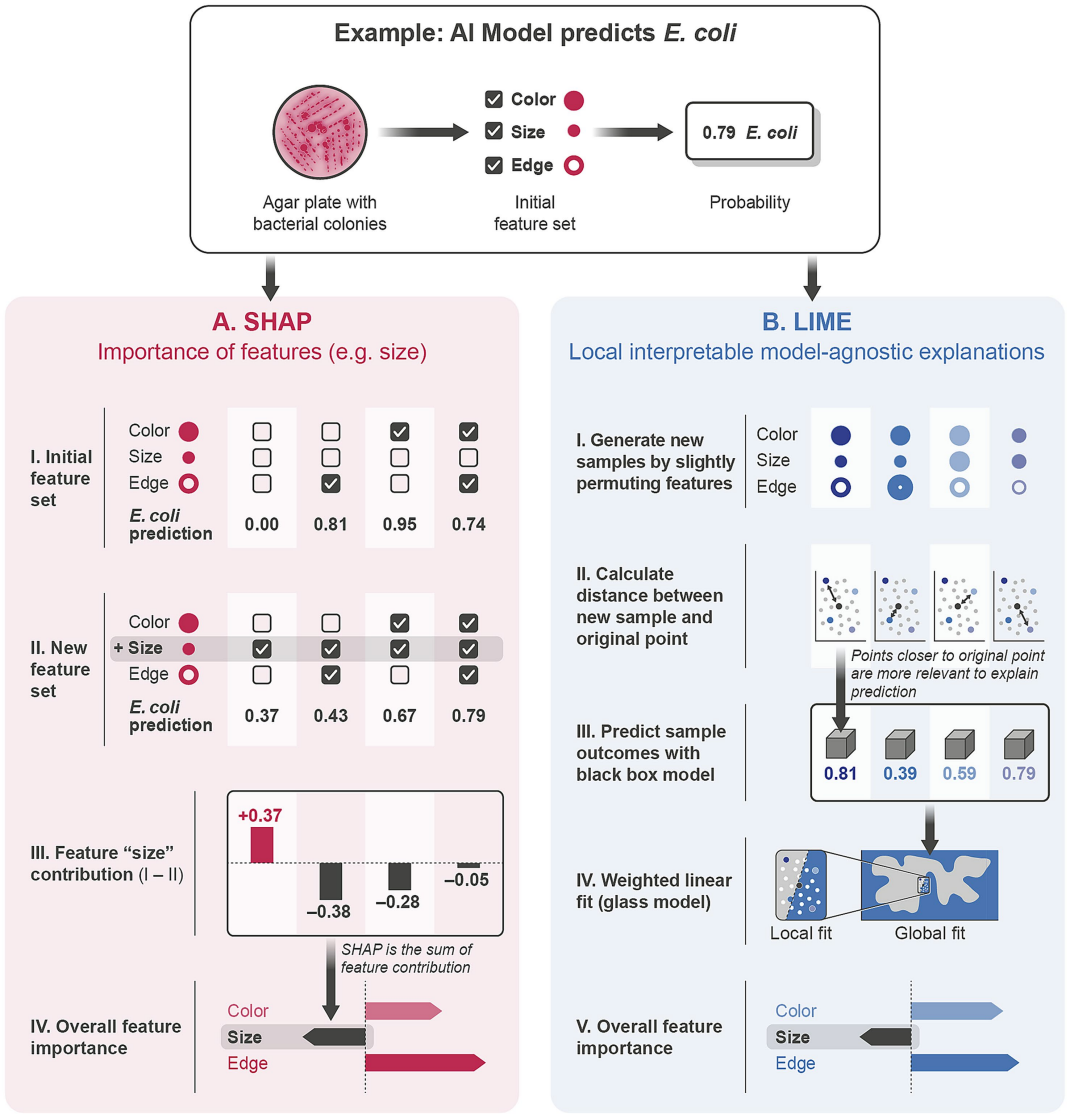
Application of explainability measures in clinical decision support systems. Patient data, collected from clinical records or diagnostic tests, serve as inputs for an AI predictive model. The AI model processes these inputs and generates predictive outcomes. To ensure clinical utility, explainability measures are applied, elucidating feature importance through interpretable visualizations, such as bar charts displaying importance scores of different clinical parameters (e.g., biomarkers, symptoms, demographics). Crucially, validation of the model includes both internal (local) validation to assess performance consistency within the development dataset and external validation using independent datasets or clinical centers, enhancing generalizability and reliability. The resulting interpretable predictions facilitate informed decision-making by physicians, who engage in iterative feedback loops, refining the AI model through practical insights and ongoing validation processes.

*Post-hoc explanations.* For more complex models, such as neural networks or ensemble models, post-hoc explanation methods are employed to clarify how predictions are made after the model has been trained. Tools like SHAP (44) and LIME [Local Interpretable Model-agnostic Explanations, (45)] quantify the influence of individual features on model predictions and visually present these insights. These methods do not alter the model itself but provide an understandable breakdown of its decision-making process (Figure 3).

*Hybrid Architectures.* Hybrid AI architectures combine symbolic AI, which uses explicit rules and logical reasoning to support interpretability, with sub-symbolic AI like deep learning, which learns patterns from data and makes accurate predictions. For example, in medical microbiology, a hybrid AI could use a deep neural network to analyse images of bacterial colonies on agar plates, while a rule-based system interprets the findings by matching visual traits (e.g., round shape, yellow colour) to known species. This approach allows models to maintain interpretability while benefiting from the high performance of deep learning (29).

*Selection of suitable explainability methods.* Selection of suitable explainability methods generally depends on three dimensions: transparency, explanatory scope, and data modality (Supplementary Table 1). Transparency distinguishes intrinsically interpretable (“glass-box”) from post-hoc methods clarifying complex models retrospectively. Intrinsically interpretable models provide immediate transparency but may slightly sacrifice accuracy (36). Post-hoc methods, however, maintain high accuracy while restoring interpretability post-training (46). Effective interpretability further depends on matching techniques with data modalities. For instance, Gradient-weighted Class Activation Mapping (Grad-CAM) excels in visualizing medical images, highlighting relevant regions in chest radiographs; SHAP efficiently attributes feature importance in structured tabular data like antibiotic resistance datasets, and LIME-text effectively interprets clinical triage text data (47, 48). Integrating these considerations ensures robust and transparent AI implementation in infectious diseases contexts.

## Potential use cases for explainable AI

XAI significantly enhances transparency by grounding AI-generated recommendations in clinically relevant factors, promoting informed decision-making, and reducing errors and biases. This transparency is critical for managing complex conditions such as sepsis or acute kidney injury, where clear insights into AI logic can mitigate uncertainties inherent in complex pathophysiology, e.g., during sepsis (18). Although explainability cannot eliminate random data errors, it effectively identifies and reduces systematic biases, aligning AI outputs closely with clinical judgment and fostering clinician trust. XAI may also support identifying risk factors linked to specific types of infection (Supplementary Table 2).

*Clinical decision support systems (CDSS).* AI-based CDSS hold promise to enhance medical decision-making by integrating patient data, guidelines, and expert knowledge, thus providing real-time diagnostic assistance and highlighting overlooked features (23, 49). Despite their potential, regulatory challenges such as the European Union’s (EU) *In Vitro* Diagnostic Regulation (IVDR), the EU-AI-Act, and regulations from the Food and Drug Administration (FDA), as well as insufficient validation in clinical trials, currently limit routine implementation in infectious disease management. Most studies show retrospective study designs with respective limitations; for example, the predictive performance for severe respiratory infections with rule-based models reaching 95.4% accuracy (50) was derived from a single-center retrospective cohort (*n* = 485) without external validation, and explainable XGBoost algorithms effectively predicting influenza mortality with interpretable SHAP insights (51) utilized a retrospective multicenter dataset from Taiwan (*n* = 336) but lacked prospective validation. Prospective randomized controlled trials of AI-based CDSS tools are rarely done. One such example includes an improved outcome in AI-driven sepsis care demonstrated by a randomized trial (52) (*n* = 142) across two intensive care units but still limited to a single healthcare center.

*Epidemiology and clinical risk assessment.* Traditional epidemiological models have been substantially enhanced by AI approaches by allowing the integration of complex datasets for improved forecasting, contact tracing, and outbreak management (8, 53). Examples include Naïve Bayes prediction of COVID-19 outcomes (54) (retrospective analysis of countries’ aggregated data, limited by data quality variability across regions) and ensemble models accurately forecasting Measles outbreaks by transparently prioritizing risk factors (55) (using US county-level surveillance data trained on just 2 years and tested on one). Additionally, wastewater viral surveillance correlated with clinical Mpox infections demonstrates reliable AI-based monitoring (56) (study from a single Chinese hospital with *n* = 283 total wastewater samples). In clinical settings, AI rapidly processes extensive data for effective risk stratification as demonstrated by high-accuracy emergency triage models (57) (single-center retrospective study, *n* = 276,164 patients) and predictive models for COVID-19 ICU admissions (58) (used MIMIC-III database with no external validation) that emphasize the necessity of external validation, explainability for generalizability and trust.

## Trust in AI: transparency and explainability

Various aspects contribute to mistrust issues in AI applications. Firstly, the data sources used to train the various models are often biased or sometimes unknown. Secondly, the rapid evolution of this technology has generated numerous predictive AI models, and versions thereof, with increasingly complex, hard to comprehend architectures. The increasing number of models is a problem. It is very hard to follow the versioning of the existing models. From a diagnostician’s point of view, and with respect to approval and QC, knowledge of versions (and the differences to the previous one) is needed. While often effective in terms of predictive accuracy, these sophisticated models typically lack transparency, functioning as “black box” systems whose internal decision-making processes remain opaque (59). Such opacity contributes significantly to skepticism and mistrust among clinicians, hindering wider adoption into routine clinical practice (Figure 1). Transparency has emerged as a fundamental requirement for AI adoption in healthcare, particularly in life-critical clinical decision-making scenarios. Mindful and ethically responsible usage and deployment of data and AI algorithms require careful attention to interpretability and fairness by proactively addressing biases and ensuring accountability in clinical settings (60, 61).

*Open, quality-controlled data.* For almost all commercial LLMs, the data source used for training is not transparently disclosed. For medical applications, most publications use primarily retrospective datasets from single institutions, thus introducing potential biases related to geography, specific patient populations, and institutional practices. This becomes particularly evident for AMR data, where prevalence data is not equally well documented (62) or during the COVID-19 pandemic, where molecular diagnostics testing and surveillance rates were significantly lower in high-income countries compared to low- and middle-income countries (63). Data used for AI applications should follow minimal quality standards (64).

*Performance metrics.* A broad range of performance metrics can be used to compare models. The info box summarizes the key metrics commonly used (Appendix). Metrics allow us to quantify how closely predictions match the real-world, thereby providing a basis for trust in the model. For binary classification tasks, e.g., predicting whether a bacterial isolate is susceptible or resistant to an antibiotic, common metrics include sensitivity (true positive rate, or recall), specificity (true negative rate), precision (positive predictive value of a “positive” prediction), and overall accuracy. These values are derived from a contingency table (confusion matrix) of predicted vs. actual outcomes, much like the evaluation of laboratory diagnostic tests in clinical microbiology (65).

In assessing classifier performance, a receiver operating characteristic (ROC) curve is often used to visualize the trade-off between sensitivity and 1–specificity across various decision thresholds. The area under the ROC curve (AUC) provides a single summary measure of discriminative ability. An AUC of 0.5 indicates a 50/50 chance, whereas an AUC of 1.0 indicates a perfectly performing classifier (66). For continuous predictions (regression tasks) such as estimating an exact minimum inhibitory concentration (MIC) from genomic data, different metrics apply. Instead of true/false positives, these models are judged by how close their numeric predictions are to the true values by using error measures like mean squared error (MSE) or mean absolute error, and sometimes by reporting the fraction of predictions within an acceptable range of the actual value (67). Such metrics help determine if an AI model’s continuous output, e.g., a predicted MIC is consistently near the ground truth.

Performance metrics like sensitivity, specificity, precision, accuracy, and AUC quantify AI model reliability, indicating areas needing improvement. High sensitivity and precision in antibiotic resistance classifiers increase trust in resistant strains being rarely missed, and calls being accurate. However, metrics can mislead if derived from small, biased, or unbalanced datasets, which emphasizes the necessity of external validation on diverse, representative data to ensure clinical utility and fairness (65).

## Ethical and regulatory aspects of explainable AI

Integrating AI into clinical practice involves navigating ethical and regulatory challenges. Ethical considerations focus heavily on transparency, fairness, and autonomy. Regulations such as the EU’s General Data Protection Regulation (GDPR) mandate clear disclosure of automated decision-making, protecting patient autonomy and ensuring informed consent (68). However, the opacity of commercially developed AI datasets complicates transparency and limits comprehensive bias assessments. Concepts like Lipton’s “contestability” and Ploug and Holm’s advocacy for clear, actionable patient information underscore the ethical necessity of empowering patients to challenge and engage meaningfully with AI-driven clinical recommendations (69). Ensuring fairness and robustness, through representative and openly accessible datasets is crucial to mitigate biases that could disproportionately impact vulnerable populations and exacerbate health disparities during infectious diseases outbreaks (29).

Regulatory frameworks for AI in healthcare further emphasize the need for explicit standards regarding transparency and explainability. Authorities like the FDA and the EU’s Medical Device Regulation (MDR) currently provide limited guidance on AI interpretability, which complicates the deployment of trustworthy systems. Initiatives such as the FDA’s Total Product Lifecycle framework are evolving to support ongoing validation and monitoring, yet regulatory ambiguity persists (70). Transparent AI systems are essential to enable clinicians to clearly communicate model details and safeguard informed consent and patient autonomy (71).

In infectious diseases management, explicit regulatory standards and rigorous ethical compliance are especially critical. AI-driven predictions significantly impact both individual patient management and public health responses, which is why transparency is important to prevent clinical errors and alert fatigue that may undermine trust, as demonstrated by performance issues in the Epic Sepsis Model (72). Robust, transparent, and ethically sound AI ensures effective surveillance, equitable resource allocation, and improved outcomes, especially in managing infectious diseases emergencies (29, 71).

## Limitations

There are a few limitations with this narrative review. As a narrative review rather than systematic review, our study selection was guided by targeted searches rather than a comprehensive protocol. Our review primarily focused on English-language publications, which may underrepresent contributions from non-English speaking regions. We did not apply formal quality assessment tools to the reviewed studies, though we have highlighted methodological strengths and limitations where relevant. The current evidence base consists largely of retrospective, single-center studies with limited external validation, reflecting the early developmental stage of XAI in clinical microbiology and infectious diseases. Future systematic reviews with formal quality appraisal will be valuable as the field matures.

## Future directions

Transparency mandated by regulatory frameworks such as GDPR (EU) and Health Insurance Portability and Accountability Act (HIPAA, US) highlights the importance of AI explainability in healthcare data management. Balancing interpretability and predictive performance become increasingly complex as AI models evolve but remain pertinent for clinical acceptance and patient safety (73).

Ensuring robustness (consistency despite data variability) and fairness (equitable model performance across diverse populations) is vital for user trust and reliability. Tailored explanations for clinicians, patients, and healthcare administrators through intuitive and privacy-preserving interfaces can enhance AI usability and acceptance significantly. A strategic shift towards inherently transparent, integrated models embedded directly within clinical workflows can reduce skepticism, support informed clinical decisions, and ultimately enhance patient outcomes. Such ethically sound AI frameworks require interdisciplinary collaboration involving clinicians, data scientists, ethicists, and regulatory bodies.

Five steps are recommended to implement XAI: (i) *Curate*. Create structured, high-quality datasets, e.g., MALDI-TOF spectra linked to resistance phenotypes. (ii) *Clarify.* Use interpretable models, e.g., SHAP with gradient-boosted trees for predicting antimicrobial resistance (e.g., against carbapenems). (iii) *Validate.* Prospectively test models in clinical routines, e.g., AI-driven early sepsis diagnosis in emergency units. (iv) *Train.* Educate specialists with interactive XAI workshops, e.g., interpreting SHAP visuals for MDR tuberculosis. (v) *Comply.* Align transparently with ethical and regulatory frameworks, e.g., IVDR and GDPR for trustworthy clinical integration.

Open data initiatives are essential for equitable AI and can enable researchers, particularly those in low- and middle-income countries, to effectively develop and validate AI models. Encouraging inclusive international collaboration and data sharing can address global health disparities by ensuring AI solutions accurately represent diverse populations. Furthermore, structured education programs promoting AI literacy among healthcare professionals will be crucial for successful AI integration into clinical practice and public health systems.

## Appendix: info box

In assessing binary classifiers, it is essential to look beyond overall accuracy to metrics that reveal true performance. This is especially the case in contexts with imbalanced classes or differing costs of error. Precision (Positive Predictive Value), Recall (Sensitivity), F1 Score, and AUC-ROC together offer a nuanced view:

- Precision reveals how reliable positive predictions are.
- Recall captures the ability to detect actual positives.
- F1 Score balances these two, particularly useful when neither false positives nor false negatives are negligible.
- AUC-ROC summarizes model discrimination across all possible decision thresholds.

Combined with sensitivity, specificity, and related rates, this suite of metrics provides a robust overview of classifier behaviour, enabling informed decisions in varied application domains.

**Table 1:**

Metric	Also known as	Definition	Formula
Sensitivity (TPR)	Recall, true positive rate	Probability that the model correctly identifies actual positives. Useful for ruling out disease when negative.	TP/(TP + FN)
Specificity (TNR)	True negative rate	Probability that the model correctly identifies actual negatives. Helpful for ruling in disease when positive.	TN/(FP + TN)
False positive rate (FPR)	–	Proportion of negatives wrongly classified as positive (1-Specificity).	FP/(FP + TN)
False negative rate (FNR)	Miss rate	Proportion of positives wrongly classified as negative (1- Sensitivity).	FN/(TP + FN)
Positive predictive value, PPV	Precision	Proportion of predicted positives that are true positives.	TP/(TP + FP)
Negative Predictive Value (NPV)	–	Proportion of predicted negatives that are true negatives.	TN/(TN + FN)
Accuracy		Overall fraction of correct classifications.	(TP + TN)/(TP + FP + TN + FN)
F1 score	F-measure	Harmonic means of Precision and Recall and balances both; robust for imbalanced data.	2·(Precision · Recall)/(Precision + Recall)
AUC-ROC	ROC AUC	Area under the ROC curve; measures model’s ability to rank positives higher than negatives across thresholds.	
