# Supplementary material for: Uncloaking the black-box: the need for explainable artificial intelligence in clinical microbiology and infectious diseases applications
Source: Front Public Health. 2026 Apr 2;14:1776922. doi: 10.3389/fpubh.2026.1776922 (PMC13082983; doi:10.3389/fpubh.2026.1776922)
Supplement: Supplementary file 1 [file Table_1.pdf]

Axis	Category	Core idea	Typical algorithms / tools	Infectious diseases example
Transparency	Ante-hoc / "glass-box"	Build the logic into the model itself; every parameter is natively readable [46].	Linear & logistic regression, scorecards, shallow decision trees, Bayesian rule lists	A seven-variable logistic-regression scorecard built from hospital EHRs predicts severe COVID-19 at admission; physicians can see exactly how age, CRP and O_2 sat weights combine into the risk score.
	Post-hoc	Let a complex model run, then analyse its output to expose which inputs drove a decision [46].	SHapley Additive exPlanations (SHAP), Local Interpretable Model-agnostic Explanations (LIME), counter-factuals, Integrated Gradients, Grad-CAM	A gradient-boosted ensemble that flags acute lower-respiratory infection in Ethiopian children uses SHAP bar-plots to reveal malnutrition and indoor-smoke exposure as the top two drivers for every alert.
Scope	Local explanations	Zoom in on a *single* prediction; approximate the neighbourhood to show why *this* result occurred [45].	LIME, SHAP local values, counter-factual "what-if"s, Grad-CAM heat-maps	LIME perturbs a positive dengue test case and shows that lethargy, retro-orbital pain and travel history were decisive, helping clinicians validate an otherwise unexpected call.
	Global explanations	Describe the model's behaviour *overall* across the full dataset; often also capable of local detail. [44].	SHAP summary plots, permutation importance, feature-attribution averaging, TreeExplainer	An Mpox classifier (LightGBM, 89 % accuracy) ranks fever, skin lesions and lymphadenopathy as the three most influential symptoms in the complete cohort, guiding public-health triage policy.

Data modality	Tabular	Handle structured rows & columns; explanations often visualised as bar-charts or summary beeswarms[44, 45].	SHAP (Tree/Kernel), LIME-tabular, feature importance in random forests	Nationwide surveillance tables for antibiotic resistance in *E. coli* are interpreted with Kernel-SHAP, pinpointing prior quinolone use and recent hospital stay as the strongest resistance predictors.
	Image	Map pixels to relevance; visual overlays highlight regions that drove the prediction [47, 48].	Grad-CAM/Grad-CAM++, DeepSHAP, occlusion maps, LIME-image	Grad-CAM on a CNN that detects TB in chest X-rays lights up apical opacities, letting radiologists verify the model's focus on pathologically plausible regions.
	Text / sequence	Trace how words, n-grams or tokens influence NLP decisions; may reuse attention or build local surrogates[47, 48].	Attention roll-out, SHAP-text, LIME-text, Integrated Gradients for transformers	A BERT-based triage bot for influenza-like illness highlights cough-duration and fever keywords, helping call-centre nurses justify automated risk flags to patients.

Supplementary Table 1. Taxonomy of explainable AI methods by transparency, scope, and data modality, illustrated with examples from infectious-diseases contexts.
