# Supplementary material for: Uncloaking the black-box: the need for explainable artificial intelligence in clinical microbiology and infectious diseases applications
Source: Front Public Health. 2026 Apr 2;14:1776922. doi: 10.3389/fpubh.2026.1776922 (PMC13082983; doi:10.3389/fpubh.2026.1776922)
Supplement: Supplementary file 2 [file Table_2.pdf]

Pathogen	Important risk factors	AI model used (Performance Metrics)	Explainability measures	Outcome/Diagnostic improvement	Reference
Acinetobacter baumannii	(i) Physiological parameters of cardio-respiratory function, such as carbon dioxide levels and respiratory rate, (ii) metabolic disturbances such as lactate and acidosis and (iii) past administration of antibiotics.	XGBoost (AUROC= 0.62)	Feature importance	Infection is more likely to occur in individuals with respiratory failure, higher lactate levels and acidosis, as well as individuals who have misused antibiotics.	[74]
Clostridioides difficile	Age, hypotension, initial treatment, onset, PCR cycle threshold, number of prior episodes, antibiotic exposure, fever, hypotension, pressors, leukocytosis, creatinine, lactate	Deep neural network (severity AUROC = 0.823)	SHapley Additive exPlanations (SHAP)	Age and presence of hypotension are significant risk factors, older patients are more likely to experience severe outcomes from *C. difficile* infection (CDI).<br>Lower fecal organism burden, history of prior CDI episodes are more likely to develop recurrent infections.	[75]

				Elevated WBC counts and prolonged exposure to antibiotics linked to increased risk.	
Helicobacter pylori	surface-enhanced Raman scattering (SERS) spectra	Adaptive boosting (AdaBoost), Bootstrap aggregating (Bagging), eXtreme Gradient Boosting (XGB), Gradient Boosting, LGBM, and Random Forest) (discrimination accuracy > 95% for all)	Local Interpretable Model-agnostic Explanations (LIME)	Non-invasive detection of *H. pylori* through SERS spectra analysis of gastric fluid is a novel method that complements existing *H. pylori* detection techniques.	[76]
Clonorchis sinensis	raw fresh fish consumption, frequency of raw fresh fish consumption, elevation, and water distance	Gradient boosting machine (GBM) (AUPRC = 0.997)	SHAP	Infection more likely to occur in people who consume raw fresh fish frequently (farmers more likely to contract this infection), people aged 29 and above.	[77]
Mpox virus (MPXV)	Differentially expressed genes (DEGs) *TXNRD1*, *CCNB1*, *BUB1*, *CDC20*, *BUB1B*, and *CCNA2*	Random Forest (AUC > 0.7)	Feature importance	Altered expression of the identified DEGs may increase the risk for severe outcomes	[78]
Hepatitis C virus (HCV)	Intake of non-steroidal anti-inflammatory drugs (NSAIDs), opioids, patient age, measures of healthcare	Logistic Regression (Precision=31%), Gradient Boosted Trees	Feature importance (Gini index)	Two-fold increase in the risk of developing HCV infection if taking NSAIDs.	[79]

	utilization	(Precision=87%), Stacked ensemble (Precision=97%)		Risk of HCV is higher in patients aged 50 to 60, with relative risk of 1.4 compared to those aged between 20 and 40 years.<br><br>Patients with a history of intravenous drug use had a five-fold increase in the risk of HCV	
Trypanosoma cruzi	Living in a house made of wood or wattle or daub, age, being bitten by the kissing bug, sex, family history of Chagas disease, living in an area infested by triatome bugs, patients' ? relation to an affected family member	LightGBM, XGBoost, Catboost, and Random Forest, Adaboost (AUROC =0.772)	SHAP, Random permutation	Chagas disease more likely to occur in older people, people with a family history of chagas disease, people who were bitten by triatomine bugs	[80]
Mycobacterium tuberculosis	IP-10, IL-2, IFN-γ, TNF-α, IL-15, IL-17, CCL3, and CCL8 (Cytokine biomarkers)	Random Forest (ROC > 80%)	Feature Importances	Patients with higher levels of these biomarkers may be more likely to develop active TB infection	[81]
Talaromyces marneffei	Septic shock and respiratory failure, uric acid, urea, platelets, and the AST/ALT ratios	XGBoost (Training data: AUC=97%, Testing data: AUC=90%)	SHAP	Deceased patients have higher levels of AST and a higher AST/ALT ratio compared to survivors. The AST/ALT ratio was noted to be 3.07 in patients who died versus 1.96 in those who survived. Patients with higher levels of urea, uric acid etc. are more likely to have a worse outcome	[82]
Candida	Invasive *C. glabrata* vs non-invasive *C.*	Random Forest	Permutation	Patients undergoing gastrointestinal	[83]

glabrata	*glabrata*: central venous catheters, intensive care unit stay, and gastrointestinal operations<br>Invasive *C. glabrata* vs all other invasive Candida species: renal disease and medications like diabetes therapeutics, cholesterol medications, and antiarrhythmics		Importances	operations, being in the intensive care unit, having renal diseases or a central venous catheter need to be monitored closely as they are associated with a higher likelihood of developing invasive candidiasis.	
Pseudomonas aeruginosa and Klebsiella pneumoniae	Age, intake of high alert medications, lower maximum decrease in respiratory rate	XGBoost, LightGBM, CatBoost, Random Forest, Support Vector Machine (Accuracy > 80%, AUROC > 80% and AUPRC > 80% for all)	SHAP	Higher risk of mortality for older patients, patients who use a greater number of high-alert medications, patients with lower maximum decrease in respiratory rate.	[84]
Staphylococcus aureus, Escherichia coli, K. pneumoniae	Mass spectrum	LightGBM (AUROC: 0.80 for *S. aureus and 0.74* for *E. coli),* MLP (AUROC: 0.74 for *K. pneumoniae*)	SHAP	Prediction of resistance associated changes in the antimicrobial protein. Streamlining of antibiotic regimen. Promotion of antibiotic stewardship	[16]

Supplementary Table 2. A selection of case studies in infectious diseases with different leading pathogens where explainable AI was used for the prediction of risk factors.
